# Impact of a Postintensive Care Unit Multidisciplinary Follow-up on the Quality of Life (SUIVI-REA): Protocol for a Multicenter Randomized Controlled Trial

**DOI:** 10.2196/30496

**Published:** 2022-05-09

**Authors:** Diane Friedman, Lamiae Grimaldi, Alain Cariou, Philippe Aegerter, Stéphane Gaudry, Abdel Ben Salah, Haikel Oueslati, Bruno Megarbane, Nicolas Meunier-Beillard, Jean-Pierre Quenot, Carole Schwebel, Laurent Jacob, Ségloène Robin Lagandré, Pierre Kalfon, Romain Sonneville, Shidasp Siami, Aurelien Mazeraud, Tarek Sharshar

**Affiliations:** 1 Raymond Poincaré Hospital Versailles Saint-Quentin-en-Yvelines Garches France; 2 U1018 Université Versailles Saint Quentin en Yvelines-INSERM Unité 1018 Groupe Interrégional de Recherche Clinique er d'Innovation Île-de-France France; 3 Cochin Hospital Assistance-Publique Hôpitaux de Paris Université de Paris Paris France; 4 Louis Mourier Hospital Assistance-Publique Hôpitaux de Paris Université de Paris Colombes France; 5 Louis Pasteur Hospital Chartres France; 6 Saint-Louis Hospital Assistance-Publique Hôpitaux de Paris Université de Paris Paris France; 7 Lariboisière Hospital Assistance-Publique Hôpitaux de Paris Université de Paris Paris France; 8 Institut National de la Santé Et de la Recherche Médicale (INSERM), Centre d'Investigation Clinique 1432, Module Epidémiologie Clinique, CHU Dijon Bourgogne, France; Dijon France; 9 Délégation à la Recherche Clinique et à l'Innovation (DRCI), Unité de Soutien Méthodologique à la Recherche, CHU Dijon Bourgogne, France Dijon France; 10 François Mitterrand University Hospital University of Burgundy Dijon France; 11 Albert Michallon Hospital Grenoble France; 12 Georges Pompidou Hospital Assistance-Publique Hôpitaux de Paris Université de Paris Paris France; 13 Bichat Hospital Assistance-Publique Hôpitaux de Paris Université de Paris Paris France; 14 Sud-Essonne Hospital Etampes France; 15 GHU-Paris Psychiatrie & Neurosciences Sainte-Anne Hospital Université de Paris Paris France

**Keywords:** critical illness, Post-ICU syndrome, Mortality, cognitive impairments, cognition, quality of life, patients, intensive care, post-traumatic, post intensive care

## Abstract

**Background:**

Critically ill patients are at risk of developing a postintensive care syndrome (PICS), which is characterized by physical, psychological, and cognitive impairments and which dramatically impacts the patient’s quality of life (QoL). No intervention has been shown to improve QoL. We hypothesized that a medical, psychological, and social follow-up would improve QoL by mitigating the PICS.

**Objective:**

This multicenter, randomized controlled trial (SUIVI-REA) aims to compare a multidisciplinary follow-up with a standard postintensive care unit (ICU) follow-up.

**Methods:**

Patients were randomized to the control or intervention arm. In the intervention arm, multidisciplinary follow-up involved medical, psychological, and social evaluation at ICU discharge and at 3, 6, and 12 months thereafter. In the placebo group, patients were seen only at 12 months by the multidisciplinary team. Baseline characteristics at ICU discharge were collected for all patients. The primary outcome was QoL at 1 year, assessed using the Euro Quality of Life-5 dimensions (EQ5D). Secondary outcomes were mortality, cognitive, psychological, and functional status; social and professional reintegration; and the rate of rehospitalization and outpatient consultations at 1 year.

**Results:**

The study was funded by the Ministry of Health in June 2010. It was approved by the Ethics Committee on July 8, 2011. The first and last patient were randomized on December 20, 2012, and September 1, 2017, respectively. A total of 546 patients were enrolled across 11 ICUs. At present, data management is ongoing, and all parties involved in the trial remain blinded.

**Conclusions:**

The SUVI-REA multicenter randomized controlled trial aims to assess whether a post-ICU multidisciplinary follow-up improves QoL at 1 year.

**Trial Registration:**

Clinicaltrials.gov NCT01796509; https://clinicaltrials.gov/ct2/show/NCT01796509

**International Registered Report Identifier (IRRID):**

DERR1-10.2196/30496

## Introduction

### Background and Rationale

In the last 2 decades, mortality has significantly decreased in the intensive care unit (ICU) [1]. However, the mortality rate at 1 year after ICU discharge remains high, ranging from 10% to 30%, according to age and severity of critical illness [2-5]. In addition, ICU survivors often develop physical, psychological, and cognitive impairments, which have been grouped under the term postintensive care syndrome (PICS) [6,7]. The incidence of post-ICU complications depends on various factors, including the patient’s pre-existing medical history, age, critical illness severity, as well as ICU and post-ICU care [8]. Because PICS is a dynamic process, its incidence changes according to the time of its assessment after ICU discharge. Physical disabilities are reported in about 14%-39% of patients at 1 year after ICU discharge and are mainly related to an ICU-acquired weakness [3,9-13]. Post-ICU psychological disorders include anxiety, depression, and posttraumatic stress syndrome (PTSD), which can affect from one-fifth to two-thirds of patients [3,14-18]. They result in an increased risk of suicide [19]. Concerning cognitive functions, 30%-90% of patients will complain of impaired memory, attention, concentration, and speech fluency [8,20-23]. They are more frequent in patients with pre-existing cognitive dysfunction and among those who have severe critical illness or who have developed delirium during their ICU stay [22]. PICS has a dramatic impact on a patient’s post-ICU trajectory, with an increased rate of mortality and rehospitalization and decreased return to home and work; it therefore profoundly affects a patient’s quality of life (QoL) [7]. Indeed, 6-month to 1-year post-ICU mortality ranges from 10% to 45%, according to age and severity and cause of critical illness [3,4,24-26]. Nearly 5%-10% of patients are readmitted to an ICU within the first year after their ICU discharge [4,27,28]. The 6-month to 1-year post-ICU QoL is significantly lower than age and sex-matched populations, with an impairment of physical, mental, and social domains [24,25,29]. QoL has been reported as being reduced by 29%-63% [30,31].

Because of its medical, social, and economic burden, PICS has been identified by the community of ICU physicians as a research priority [7]. There have been randomized controlled trials (RCTs) on post-ICU interventions, but the type of intervention, endpoints, time frame, and populations have varied.

Briefly, a rehabilitation program has been shown to improve physical [32] and psychological [4] status and increase patient satisfaction, but it has not ameliorated health-related QoL (HRQoL) [33]. A systematic review of RCTs indicated that post-ICU follow-up models focusing on psychological or medical management interventions were associated with fewer PTSD symptoms [34]. RCTs on care coordination have shown that neither a nurse-led post-ICU program in critically ill patients nor primary care physician–led follow-up in patients with sepsis was beneficial [24,35].

At the time of the design of our RCT (ie, 2010), there were a limited number of RCTs that focused on the benefit of post-ICU care coordination, but none integrated a social follow-up. Because of the interdependency of its domains, we hypothesized that a medical, psychological, and social follow-up would be more appropriate for mitigating PICS. We carried out a multicenter RCT to determine whether a post-ICU multidisciplinary follow-up would improve QoL at 1 year and would then also improve physical, psychological, cognitive, and social status and reduce mortality and medical requirements (ie, hospitalization, outpatient consultations).

### Objectives

The primary objective is to assess the impact of medical, psychological, and social follow-up on death and HRQoL at 1 year after discharge from ICU.

The secondary objectives are to assess the benefit of a post-ICU follow-up on muscle strength, functional capacities, cognitive abilities, and psychological state, as well as on social and professional reintegration.

### Trial Design

The SUIVI-REA trial is an open multicenter parallel group RCT comparing a program of medical, psychological, and social follow-up with standard care in patients 1 year after ICU discharge.

## Methods

### Study Setting

A total of 11 centers, including 9 general hospitals and 2 university hospitals, participated in this study. Factors determining center participation were a capacity to include patients, and the availability and willingness of psychologists, social workers, and physicians to implement post-ICU consultations. All participating centers had previously participated in clinical trials. Training on study procedures was provided to all participating staff members. Documents required for the study, including the study protocol and management guidelines, were available in each participating ICU.

### Eligibility Criteria

Adult patients were eligible if they (1) lived in an area near the participating center; (2) had required mechanical ventilation (MV) for more than 3 days; (3) had a life expectancy greater than 1 year (defined by a McCabe score <2 and the absence of metastatic cancer); (4) were enrolled with a general practitioner; (5) were affiliated to the social health care system; and (6) had given their written informed consent. Duration of MV of at least three days was selected for patients with severe critical illness. A general practitioner was mandatory because we believe he/she should be involved in the post-ICU follow-up program. Proximity to a participating hospital was to facilitate attendance at post-ICU consultations.

Patients were excluded from the trial if they (1) had been hospitalized in an ICU in the previous year; (2) were followed for a pre-existing chronic myopathy; (3) had been admitted for serious burns, severe brain injury, suicide, or self-induced poisoning; (4) had a psychiatric disorder or dementia; (5) were under guardianship; (5) did not speak fluent French; (6) were homeless; and (7) were pregnant. These criteria were established to exclude those not benefiting from a specific follow-up and those who were unable to give their consent or to follow the post-ICU program. Patients with chronic myopathy were excluded because 1 participating center already had in place an organized, specific long-term multimodal follow-up for this condition.

All patients from one of the participating ICUs were screened for eligibility by ICU physicians before hospital discharge. The reasons for nonrandomization were collected.

### Informed Consent

Written informed patient consent had to be obtained by the investigator of the participating center. A copy of the consent form was given to every patient, with the investigator retaining a copy.

As observational studies have shown that patients with delirium were at risk of developing psycho-cognitive disorders [36], we decided to remove impairment of consciousness as an exclusion criterion because a post-ICU follow-up would be beneficial for these patients. In case of impaired consciousness (ie, delirium), the investigator sought written consent from the next of kin. As soon as the patient’s status allowed, written informed consent for the continuation of the research and analyses of the data was obtained. There was no additional consent for ancillary studies.

### Interventions

#### Explanation for the Choice of Comparators

At the time of the trial design, there was no recommendation for post-ICU follow-up, in terms of both type and rate of consultations. Therefore, the comparator did not differ from the current practice and “no post-ICU follow-up” was used to control the intervention.

#### Intervention Description

Patients were included at time of their discharge from the ICU either to home or to another department of the same or another hospital. By convention, day 1 corresponded to the date of inclusion. The randomization was performed after the baseline assessment at the time of inclusion. The last consultation was planned at 12 months, after the simple blinded collection of the primary endpoint.

In both therapeutic groups, patients’ medical, psychological, and social scores and questionnaires were assessed at the time of inclusion and at 12 months, to evaluate whether the characteristics of the 2 therapeutic groups were comparable at baseline and to determine the respective course of post-ICU discharge impairments in both groups. The scores and questionnaires were completed by the patient alone or with the help of the research assistant. If the patient was included in the control group, the test results were sealed for disclosure at the end of the study. Test results for intervention group patients were passed to the multidisciplinary team to avoid repeating the tests.

The patients from the control group had no additional post-ICU consultation. In the intervention group, patients received a multidisciplinary consultation within the first weeks after inclusion, at 3 months, and (if necessary) at 6 months. This was a consultation with a physician, a psychologist, and a social worker. Both the physician and social worker were from the ICU participating in the trial. The ICU-referring psychologist was active in 9 centers. A psychologist was specifically recruited for performing the follow-up in the remaining 2 centers. The same multidisciplinary team was used for follow-up in each patient.

The ICU physician consultation comprised (1) the collection of information about the current treatment, weight, vital signs, comorbidities, and symptoms; (2) the date and cause of readmission at the hospital; (3) standardized general examination; (4) functional status using the Medical Research Council (MRC) sum score for assessing muscle strength and the Barthel index and Instrumental Activities of Daily Living (IADL) scores for assessing disability; and (5) cognitive status using the Minimal Mental State (MMS) scoring system. It was recommended that the participant was followed by the same ICU physician throughout follow-up. The ICU physician could prescribe a paraclinical exploration or a treatment, but it was recommended that they referred to the patient’s general practitioner, except in the case of an emergency.

The consultation with the psychologist consisted of the collection of the Hospital Anxiety and Depression Scale (HADS) and Impact Event Scale-Revised (IES-R) scores for assessing PTSD as well as an interview during which the participant reported any psychological difficulties. The consultation with the social worker consisted of the collection of the social questionnaire response and an interview during which the patient reported about their QoL (Euro Quality of Life-5 dimensions [EQ5D]), social and professional difficulties, and needs.

In total, the medical, psychological, and social consultations took 2 h and 30 min. They were followed by a meeting between the ICU physician, psychologist, and social worker to discuss the participant’s status and requirements and to write a summary report to forward to the general practitioner.

### Criteria for Discontinuing or Modifying Allocated Interventions

Any participant could discontinue participation in the research at any time for any reason. The investigator could temporarily or permanently discontinue one’s participation in the research for any reason that affected the participant’s safety or was in the best interests of the participant. In the event of premature termination of the research, or withdrawal of consent, data collected prior to the premature termination could be used. The reasons for discontinuing participation in the research were to be registered in the participant’s file.

### Strategies to Improve Adherence to Interventions

The participating teams were informed monthly of the course of the study and reminded of the main elements of the trial. The research technician at each center organized consultations for intervention patients at 3, 6 (if needed), and 12 months. Patients were reminded of these consultations 15 days beforehand. Control patients were called at least once by the research technician to remind them of their 12-month consultation, which was planned at time of their ICU discharge.

### Relevant Concomitant Care Permitted or Prohibited During the Trial

For deontological reasons, the patients of both groups continued to be followed by their general practitioner or specialist physician, but they were allowed to see a new physician, a physiotherapist, or psychologist. The purpose of the trial was to determine whether a post-ICU follow-up improved standard patient care.

### Provisions for Posttrial Care

In France, the research sponsor insurance offers a subsequent period of insurance 10 years from the end of the research. Consequently, in the event of poststudy damage to participants related to their participation in the research, the complaint would be admissible whenever it occurred during this period.

### Outcomes

The primary outcome was QoL at 12 months. The QoL was assessed using the EQ5D and by telephone by a blinded investigator. The patient was asked not to disclose to which group they were randomized. The EQ5D is a standardized self-completed instrument for measuring generic HRQoL. The Euro Quality of Life-5 dimensions-5 Levels (EQ5D-5L) comprises 5 dimensions: mobility, self-care, usual activities, pain/discomfort, and anxiety/depression. Each dimension has 5 problem levels: none, slight, moderate, severe, and extreme. Finally, the weighted sum of the responses obtained provides a cardinal measure (between 0 for death and 100 for the total absence of a problem), which is also suitable for medicoeconomic evaluation [37]. The EQ5D has been used in various post-ICU follow-up studies [30,35,38-41]. The secondary outcomes were mortality, cognitive, psychological, and functional status; social and professional reintegration at 1 year; as well as the rate of rehospitalization and outpatient consultations within the first year (Table 1).

**Table 1:** Research timeline for each participant.

Timepoint	Inclusion	ICU^a^ discharge	3 months	6 months	12 months
Consent collection	Both groups				
Pursue consent collection		Both groups	Both groups	Both groups	
Demographics, medical history, critical illness, and ICU stay characteristics	Both groups				
Collection of clinical data	Both groups	Follow-up group	Follow-up	Follow-up	Both groups
Standard biological tests	Both groups		group	group	Both groups
Adverse events		Follow-up group	Follow-up	Follow-up	Both groups
Final assessment of main outcome					Both groups
Final assessment of secondary outcomes					Both groups

^a^ICU: intensive care unit.

### Sample Size

At the time of the study design, other studies indicated that 10% of patients discharged from ICU died within the first year and 40% had a moderate to severe impairment on at least one dimension of the EQ5D [32,41].

Therefore, we estimated that at least 50% had a very poor outcome, combining death and severe to extreme impairment of at least one EQ5D dimension. [23]. The study was then powered to detect a decrease from 50% to 37% of patients with very unfavorable outcome with a power of 80% and a 2-sided 5% alpha risk, assuming this rate would be 50% in the control arm. Accordingly, the sample size was 249 patients per group. We anticipated that 20% of the patients would be lost to follow-up, so the sample size was increased to 300 per arm. The study therefore initially planned to enroll a maximal sample size of 600 patients. However, as the rate of loss to follow-up has, indeed, proved to be 10%, we decided to decrease the sample size to 520 patients. Finally, 546 patients were included. Interestingly, recent studies on comparable populations of critically ill patients showed that there was a 1-year mortality rate of between 10% and 28% [2,3,5,8,20] and that about 60% of patients had a moderate to severe impairment on at least one dimension of the EQ5D [3,10,29]. These findings suggest that our original estimation is still appropriate.

### Recruitment

The study took place in 11 ICUs, which had been selected based on the interest expressed by local teams in post-ICU follow-up, for their capacity to recruit patients and to handle the restraints of an RCT. A research assistant was available at every participating center to screen patients for inclusion. The steering committee met monthly. A centralized phone and email center answered participating center questions regarding patient eligibility or management during the entire trial period. A monthly newsletter was circulated, informing participating centers of the number of patients included, main study constraints, and any protocol modifications (Table 2).

**Table 2:** Number of patients included and participating centers and inclusion rate.

Evaluation	Values required
Number of patients to be included	546
Number of centers	11
Number of months	54
Number of patients per month per center	1

### Assignment of Interventions: Allocation

#### Sequence Generation

The randomization list, generated by an independent statistician, was balanced between arms. Randomization was stratified by center using a permuted block of unrevealed size randomization. As critical severity could potentially impact the rate and intensity of psychological, cognitive, and physical impairment, stratification was made on this basis.

#### Concealment Mechanism

Randomization and concealment were ensured by using a secure dedicated web-based system accessible at each study center and managed by the clinical research unit (CRU), which had no role in patient recruitment.

#### Implementation

The allocation sequence was generated by the study statistician. Patient enrollment was established by the participating center investigator.

### Assignment of Interventions and Blinding

The outcome assessor assessing the EQ5D by phone was blinded. Neither the participants nor the investigators (ie, ICU physicians, psychologists, and social workers) were blinded for patient assignment to one of the trial groups. The procedure for unblinding was not planned as the intervention was considered safe.

### Data Collection and Management

#### Plans for Assessment and Collection of Outcomes

At inclusion, baseline characteristics were systematically collected by the center investigator as follows: demographic and anthropometric data; location prior to ICU admission (community, hospital, or long-term facility); pre-existing comorbidities using Knaus, McCabe, and Charlson scores; date and time of ICU admission; and severity of critical illness at ICU admission using the Simplified Acute Physiology Score (SAPS-II) and the Sequential Organ Failure Assessment (SOFA) score [42,43]. In addition, body weight, height, vital signs, MRC sum score [9], the Barthel index [44], IADL scores [45], MMS [46], HAD-S [47], IES-R [48], and the social questionnaire were collected for all participants. Patients’ were also asked to score their QoL 3 months before ICU admission, using the EQ5D tool. The functional and psychological scores were completed by the patients, with help from the research technician. The MRC sum score and MMS were assessed by the ICU physician in charge of the patients. This assessment ensured that both the functional, cognitive, psychological, and social status and pre-ICU QoL were comparable between the 2 trial groups. Finally, blood samples were taken for standard biological tests, including blood cell count; biochemistry; and plasma levels of C-reactive protein, pre-albumin, albumin, and thyroid hormones.

At time of ICU discharge the duration of MV, need for tracheostomy, and length of ICU stay were also recorded.

Intervention group patients were seen by the ICU physician, the psychologist, and the social worker before ICU discharge (month 0) and at 3 months, and eventually at 6 months by the follow-up team. The psychologist and social worker had access to the scores and questionnaire completed by the patients at inclusion. At 3 and 6 months, the MRC sum score, Barthel index, IADL, MMS, HADS, and IES-R were collected as well as the social questionnaire and results of the biological tests.

At 12 months, the EQ5D (ie, primary endpoint) was assessed on the phone by a blinded assessor. All patients were seen by the ICU physician, psychologist, and social worker. The MRC sum score, Barthel index, IADL, MMS, HADS, and IES-R, together with the 36-item Short Form (SF-36) for assessing QoL and the social questionnaire, were collected. Standard biological tests were performed at 12 months.

The reason for failure to attend the planned consultation was recorded by the research technician via telephone. The date and cause of readmission to hospital and death were also documented. The number of consultations with the general practitioner or any other specialist was recorded.

#### Plans to Promote Participant Retention and Complete Follow-up

Each month, the participating teams were informed of the course of the study and reminded of the main elements of the trial, notably concerning the organization of the follow-up consultation.

#### Data Management

Data management and statistical analysis were performed independently of the sponsor and of investigators by the CRU (Unité de Recherche Clinique, Hôpital Ambroise-Paré, Boulogne-Billancourt, France). Data entry was performed by the investigator at enrolling sites using a web-based data entry system.

An electronic case report form (eCRF) was developed by the CRU using dedicated software (CleanWeb) to facilitate data control and monitoring. Each patient was assigned a unique study ID that was used to index the eCRF and related study documents. It captured data from each included patient.

All information required by the protocol had to be entered in the eCRFs. Data were recorded in the eCRF as and when they were obtained. Any missing data had to be coded. In-built consistency checks instantly verified the coherence of data.

Data monitoring was performed by the sponsor (Direction de la Recherche Clinique et de l’Innovation de l’Assistance Publique – Hôpitaux de Paris [DRCI AP-HP]). This project was classified as interventional with potential risks based on the AP-HP risk level classification, meaning that a high level of monitoring is necessary for determining whether centers adhere to the protocol and procedures, for checking for eCRF completeness, for ensuring patient safety (adverse event/serious adverse event), and for follow-up in accordance with the applicable regulations. A clinical research associate (CRA) appointed by the sponsor is responsible for timely completion of the study and for collecting, documenting, recording, and reporting all handwritten data, in accordance with the standard operating procedures applied within the DRCI APHP and in accordance with Good Clinical Practices as well as the statutory and regulatory requirements. During these visits, the following elements were reviewed:

- Written consent.
- Safety and rights of participants being protected.
- Compliance with the study protocol and with the procedures defined therein.
- Quality of data collected in the eCRF (accuracy, missing data, consistency of the data with the “source” documents, such as medical files, appointment books, original copies of laboratory results, etc.).
- Data were authentic, accurate, and complete.

The CRA systematically checked baseline characteristics, eligibility criteria, primary outcome, and serious adverse events reported in the eCRF for all study participants. In addition, for one-third of the study population, all data reported in the eCRF were validated against a patient’s original chart. Serious adverse events and major protocol violations were reported to the DRCI APHP and Comité de protection des personnes (CPP; Ethics Committee).

At the end of the study, after clarification of discrepancies (data cleaning) and data validation, the database was frozen and transmitted to the statistician, following procedures established by the sponsor.

Each patient participated in the trial for 12 months. Premature study withdrawal was at the request of the patient or next of kin and their reasons were recorded in the eCRF and the patient’s medical file. Withdrawn patients were not replaced. However, patients who were lost to follow-up or did not receive the randomly assigned treatment were not considered to be prematurely withdrawn from the trial.

### Confidentiality

As for any clinical research supported by the AP-HP, processing of personal data complied with the methodological requirements for a clinical trial established by the French Data Protection Authority Commission Nationale de l’Informatique et des libertés Commission Nationale de l’Informatique et des libertés (CNIL) in January 2006 for biomedical research. During and after the clinical research, all collected data sent to the sponsor by the investigators (or any other specialized collaborators) were pseudonymized using only the participant’s initials. Under no circumstances the names and addresses of the participants involved had been shown. Only the participant’s initials and an encoded number specific to the study indicating the order of enrollment were recorded. Moreover, all nominal data were erased on the copies of the source files that were used for research documentation.

### Plans for Collection, Laboratory Evaluation, and Storage of Biological Specimens for Genetic or Molecular Analysis in This Trial/Future Use

No genetic or molecular analyses were planned.

### Statistical Methods

#### Statistical Methods for Primary and Secondary Outcomes

The 1-year survival rate without major deterioration in QoL (main endpoint, defined as reporting of death or a severe to extreme problem” level in 1 of the 5 dimensions studied) will be compared between both arms using a piecewise exponential model considering any censorship and the repeated nature of observations, prohibiting the use of conventional methods of analysis of censored data. This analysis will be adjusted for age and severity of critical illness (according to SOFA grading), and the center will be considered as a random effect. In addition, 2 analyses will be performed, according to age category (with cut-off at 65 years) and severity of critical illness (with a cut-off at the median value).

Binary outcomes will be analyzed using logistic regression. Absolute risk reductions will be obtained using a binomial model with identity link [49]. For time-to-event outcomes, Kaplan-Meier survival curves or cumulative incidence curves will be estimated, and the intervention effect will be analyzed using the Cox proportional hazards regression. Mixed linear regression will be used for continuous outcomes, possibly after variance-stabilizing transformation. All tests will be 2-sided at a .05 significance level.

#### Interim Analyses

We neither planned nor performed an interim analysis.

#### Methods for Additional Analyses (eg, Subgroup Analyses)

Age and severity of critical illness as predictors of poor outcomes and practice might differ between centers, so randomization was stratified by center and statistical analysis is adjusted for these factors to minimize discrepancies between therapeutic groups.

#### Methods in Analysis to Handle Protocol Nonadherence and Any Statistical Methods to Handle Missing Data

Intent-to-treat statistical analysis will be performed after all patients have completed the 1-year follow-up. Accordingly, all patients will be analyzed in the arm they were allocated to, regardless of protocol deviations. In addition, missing outcome data will be imputed. Prior to any data analysis, a detailed statistical analysis plan will be drawn up by the study statistician. There will be a comprehensive report of the statistical analysis, following the CONSORT statement recommendations. Any change in the analysis plan will be justified in this final report.

While no missing data are expected, the maximum bias method will be used for analysis of the primary outcome, replacing missing data by a success in the control arm and by a failure in the intervention arm. For secondary outcomes, missing data will be handled by multiple imputations by chained equations. A sensitivity analysis of complete cases only will be performed.

#### Plans to Give Access to the Full Protocol, Participant-Level Data, and Statistical Code

Those with direct access in accordance with the laws and regulations in force, in particular articles L.1121-3 and R.5121-13 of the Public Health Code (eg, investigators, those responsible for quality control, monitors, CRAs, auditors, and others involved in collaborating on trials), will take all necessary precautions to ensure the confidentiality of information relating to the tested drugs, the trial, and the trial participants, especially with regard to their identity and the results obtained. The data thus collected during quality controls or audits are then made anonymous.

### Oversight and Monitoring

#### Composition of the Coordinating Center and Trial Steering Committee

The steering committee is composed of DF and TS who initiated the project, the methodologist and the sponsor’s representatives (DRCI and CRU APHP) appointed for this research. The steering committee aimed at deciding during the trial the procedures to be followed, taking note of the recommendations of the independent supervisory committee. It defined the general organization and conduct of the research, and coordinated the information. It also decided the appropriate methodology to conduct for unforeseen circumstances. During the trial period it will determine and monitor the progress of the research, particularly in terms of tolerance and adverse events.

#### Composition of the Data Monitoring Committee, Its Role, and Reporting Structure

There was no Data Safety Monitoring Board (DSMB) as the intervention was considered safe for the patient.

### Adverse Event Reporting and Harms

Any adverse event occurring during the trial period was reported by participating centers via a centralized phone and email system. Serious adverse events and major protocol violations were reported to the DRCI and CPP.

### Plans for Communicating Important Protocol Amendments to Relevant Parties (eg, Trial Participants, Ethical Committees)

All substantial modifications to the protocol by the coordinating investigator were sent to the sponsor for approval. After approval was given, the sponsor obtained approval from the CPP (Research Ethics Committee) and authorization from the Agence Nationale de Sécurité du Médicament (ANSM) within the scope of their respective authorities before the amendment was implemented.

The information note and the consent form had been revised, particularly in case of a substantial amendment to the study.

### Dissemination Plans

Neither the study sponsor nor the study funder had any role in designing the trial, managing, analyzing, or interpreting the data, writing the report, or in the decision to submit the report for publication.

### Patient and Public Involvement

No patient involved.

### Availability of Data and Materials

In accordance with Good Clinical Practice: (1) the sponsor is responsible for ensuring all parties involved in the study agree to guarantee direct access to all locations where the study will be carried out, the source data, the source documents, and the reports, for the purposes of the sponsor’s quality control and audit procedures or inspections by the competent authority; (2) the investigators allow individuals in charge of monitoring and quality control to have access to the documents and personal data strictly necessary for these tasks, in accordance with the statutory and regulatory provisions in force (Articles L.1121-3 and R.5121-13 of the French Public Health Code).

The AP-HP had full access to patients’ charts and checked all data recorded in the eCRF against original charts. All information required by the protocol had to be provided in the electronic logbook and an explanation given by the investigator for any missing data.

### Ethics Approval and Consent to Participate

Methodological aspects were independently approved by the national jury of the Clinical Research Hospital Program in 2010, and the Ministry of Health confirmed funding under contract number AOM10072. The protocol and qualification of all investigators were approved by the CPP of Saint-Germain-en-Laye, France, on July 08, 2011. The CPP allowed for waiver of consent and deferred consent (number 11052). The trial was registered at Clinicaltrials.gov identifier NCT01796509 (registered on February 21, 2013).

Written informed consent of the patient had to be obtained by the investigator of the participating center. In case of impaired consciousness, the investigator sought for written consent from the next of kin. If the latter was not present, the patient could be included as deferred consent, as has been approved by the Ethics Committee, according to French law (Art L1122-1-2 du Code de la Santé Publique). As soon as the patient’s status allowed, written informed consent for the continuation of the research and analyses of the data was obtained. A copy of the consent form was given to every patient. The original copy must be retained in the investigator’s archive for a minimum of 15 years. A third copy is archived by the sponsor. Patients or the public were not involved in the design, or conduct, or reporting, or dissemination plans of our research.

## Results

The study was funded by the Ministry of Health in June 2010. It was approved by the Ethics Committee on July 8, 2011. The first and last patient were randomized on December 20, 2012, and September 1, 2017, respectively. A total of 546 patients were enrolled across 11 ICUs. At present, data management is ongoing, and all parties involved in the trial remain blinded.

The first patient was recruited on December 20, 2012, and the last patient on September 1, 2017. The study was never suspended. The assessor of the primary endpoint and study statistician remained blinded to study intervention throughout the trial. Data management is ongoing. Release of the results is planned for end of 2022.

There were 10 amendments to study protocol (Multimedia Appendix 1). All amendments were approved by investigators, study statistician, AP-HP, CPP, and ANSM.

The DRRC organized data monitoring and quality audits. Baseline characteristics, eligibility criteria, primary outcome, secondary outcomes, and serious adverse events reported in the eCRF were systematically checked against original charts for all research participants. In addition, for one-third of the study population, all data reported in the eCRF were validated against a patient’s original chart. Serious adverse events and major protocol violations were reported to the DRRC, ANSM, and CPP. The study coordinator had quarterly face-to-face meetings with the DRRC and AP-HP to monitor trial conduct according to the highest standard for protection of research participants. All randomized patients completed follow-up for the primary outcome.

Monitoring of the data has been completed. Freezing of the database and statistical analysis are planned within the next 6 months.

## Discussion

### Overview

PICS is a major public health issue, affecting more than half of critically ill patients 1 year after their discharge from an ICU [8]. It has a huge impact on QoL, affecting a patient’s personal, social, and professional life. In their Cochrane review, Schofield-Robinson et al [50] concluded that there was “insufficient evidence, from a limited number of studies, to determine whether ICU follow-up services are effective in identifying and addressing the unmet health needs of ICU survivors” and called for future studies that are “designed with robust methods (for example, randomized studies are preferable) and consider only one variable (the follow-up service) compared to standard care.” Our trial is in line with these recommendations as it is a multicenter parallel group RCT that was designed to determine whether a medical, psychological, and social follow-up improves QoL of the critically ill patient at 1 year after ICU discharge. This hypothesis was based on the fact that PICS is characterized by interdependent elements, which would be better targeted using a multidisciplinary approach. The SUIVI-REA trial is still original and relevant. Because the use of MV at a minimum of 3 days is an inclusion criterion enabling the selection of patients with severe critical illness, the results will be obtained from a representative population at risk of developing PICS. Its results could be then compared with those of ongoing trials assessing the benefit of telehealth [51], combined physical and cognitive training [52], or multidisciplinary personalized follow-up [53].

As cognitive impairment was not comprehensively assessed, it could be argued that it was not therefore managed. In 2010, when the study was designed, post-ICU cognitive dysfunctions were not identified as a major component of PICS. Moreover, we considered that compliance would have been reduced, as a cognitive assessment would have increased the length of the consultation period and the number of questionnaires patients had to complete. In our RCT, cognitive impairment was assessed using the MMS examination, which has been validated for detecting dementia and used in cohort studies of critically ill patients [20].

The program might be thought to be an assessment of physical, psychological, and social domains rather than one of multidisciplinary care. However, we felt that the multidisciplinary teams should not replace the physicians who routinely care for a patient but rather that they should contribute to patient care, notably by detecting ICU-related complications and suggesting specific management to the patient’s own doctor. Multidisciplinary teams were highly recommended to respect these deontological principles. For this reason, a concluding letter was systematically sent to the general practitioner, recapitulating the observations and propositions made by the multidisciplinary team. The impact of our intervention would therefore depend on the commitment of the multidisciplinary teams to participate in the patient’s overall care. Should our intervention not have added value to routine care, our hypothesis is that its main benefit would be the expertise of the multidisciplinary team to assess and treat ICU-related complications. In addition, the social assessment would help to personalize a patient’s care.

### Randomization Procedure

Selection biases were minimized and randomization ensured homogeneity between the 2 groups. First, the random list for allocating interventions was computer generated by an independent statistician. Randomization was centralized through a secured website using permutation blocks, the size of which was unknown to research participants. Neither the investigators nor the patients could be blinded for the intervention. It is not possible to anticipate any advantages for an intervention and what their extent might be.

Strategies have been established for limiting the loss of follow-up and to improve patient attendance at consultations, by regular telephone reminders, planning of the consultation with the patient, and organization of the patient’s home-hospital transport. Finally, amendments made to the protocol aimed to improve patient recruitment.

We neither planned nor performed an interim analysis.

### Endpoints

The primary endpoint is QoL, to be evaluated using a validated scale (ie, EQ5D). We considered QoL to be the most appropriate endpoint for evaluating both a multidimensional condition (ie, PICS) and the intervention. The EQ5D has been collected by phone by an assessor blinded to randomization. This has been a customary procedure in various clinical trials evaluating QoL as the primary endpoint [29]. The secondary endpoints are conventional and will enable us to assess the impact of the multidisciplinary follow-up on the principal dimensions of PICS.

### Strength and Limitations of the Study

- This is the first multicenter RCT that assesses whether a post-ICU multidisciplinary follow-up program based on medical, psychological, and social assessment will improve the QoL at 1 year.
- This RCT has been designed and powered for addressing this major issue, because ICU stay is associated with increased mortality and morbidity.
- The trial is based on a clinically relevant primary endpoint, that is, QoL, that considers mortality as well as ICU-induced physical, psychological, and social impairment(s).
- This trial concerns only adult patients discharged from an ICU.
- Because medical, psychological, and social assessments are time-consuming, a comprehensive neurocognitive evaluation was not feasible.
- Strategies were applied for limiting the loss to follow-up and improving assiduity for follow-up consultations.

### Conclusion

Post-ICU interventions have been little studied and to date none have been shown to be beneficial. Therefore, SUIVI-REA is designed to demonstrate the benefit of post-ICU follow-up services for mitigating PICS in a representative population of ICU-discharged patients at risk of developing PICS. By integrating adjustments to the main outcome predictors and collecting potential confounding factors, the trial will also provide original, reliable, and relevant data on the epidemiology of PICS. This will not only help in the design of further clinical trials but also enable the development of algorithms for predicting PICS [54].

## Appendix

Multimedia Appendix 1 Supplementary table: Amendments to study protocol.

## Abbreviations

ANSM: Agence Nationale de Sécurité du Médicament
AP-HP: Assistance Publique – Hôpitaux de Paris
CNIL: Commission Nationale de l’Informatique et des libertés Commission Nationale de l’Informatique et des libertés
CPP: Comité de protection des personnes (Ethics Committee)
CRA: clinical research associate
CRU: clinical research unit
DRCI: Direction de la Recherche Clinique et de l’Innovation
DSMB: Data Safety Monitoring Board
eCRF: electronic case report form
EQ5D: Euro Quality of Life-5 dimensions
HADS: Hospital Anxiety and Depression Scale
HRQoL: health-related quality of life
IADL: Instrumental Activities of Daily Living
ICU: intensive care unit
IES-R: Impact Event Scale-Revised
MMS: Minimal Mental State
MRC: Medical Research Council
MV: mechanical ventilation
PICS: postintensive care syndrome
PTSD: posttraumatic stress syndrome
QoL: quality of life
RCT: randomized controlled trial
SAPS-II: Simplified Acute Physiology Score
SF-36: 36-item Short Form
SOFA: Sequential Organ Failure Assessment
