# Correction: Impact of a Postintensive Care Unit Multidisciplinary Follow-up on the Quality of Life (SUIVI-REA): Protocol for a Multicenter Randomized Controlled Trial

**DOI:** 10.2196/47929

**Published:** 2023-04-14

**Authors:** Diane Friedman, Lamiae Grimaldi, Alain Cariou, Philippe Aegerter, Stéphane Gaudry, Abdel Ben Salah, Haikel Oueslati, Bruno Megarbane, Nicolas Meunier-Beillard, Jean-Pierre Quenot, Carole Schwebel, Laurent Jacob, Ségloène Robin Lagandré, Pierre Kalfon, Romain Sonneville, Shidasp Siami, Aurelien Mazeraud, Tarek Sharshar

**Affiliations:** 1 Raymond Poincaré Hospital Versailles Saint-Quentin-en-Yvelines Garches France; 2 U1018 Université Versailles Saint Quentin en Yvelines-INSERM Unité 1018 Groupe Interrégional de Recherche Clinique er d'Innovation Île-de-France France; 3 Cochin Hospital Assistance-Publique Hôpitaux de Paris Université de Paris Paris France; 4 Louis Mourier Hospital Assistance-Publique Hôpitaux de Paris Université de Paris Colombes France; 5 Louis Pasteur Hospital Chartres France; 6 Saint-Louis Hospital Assistance-Publique Hôpitaux de Paris Université de Paris Paris France; 7 Lariboisière Hospital Assistance-Publique Hôpitaux de Paris Université de Paris Paris France; 8 Institut National de la Santé Et de la Recherche Médicale (INSERM), Centre d'Investigation Clinique 1432, Module Epidémiologie Clinique, CHU Dijon Bourgogne, France; Dijon France; 9 Délégation à la Recherche Clinique et à l'Innovation (DRCI), Unité de Soutien Méthodologique à la Recherche, CHU Dijon Bourgogne, France Dijon France; 10 François Mitterrand University Hospital University of Burgundy Dijon France; 11 Albert Michallon Hospital Grenoble France; 12 Georges Pompidou Hospital Assistance-Publique Hôpitaux de Paris Université de Paris Paris France; 13 Bichat Hospital Assistance-Publique Hôpitaux de Paris Université de Paris Paris France; 14 Sud-Essonne Hospital Etampes France; 15 GHU-Paris Psychiatrie & Neurosciences Sainte-Anne Hospital Université de Paris Paris France

In “Impact of a Postintensive Care Unit Multidisciplinary Follow-up on the Quality of Life (SUIVI-REA): Protocol for a Multicenter Randomized Controlled Trial” (JMIR Res Protoc 2022;11(5):e30496) the authors made three clarifications.

In the sample size section of the manuscript, the passage:

Therefore, we estimated that at least 50% had a very poor outcome, combining death and moderate to severe impairment of at least one EQ5D dimension

has been corrected to:

Therefore, we estimated that at least 50% had a very poor outcome, combining death and severe to extreme impairment of at least one EQ5D dimension.

In the sample size section of the manuscript, the passage:

The study was then powered to detect a decrease from 50% to 37% of patients with very unfavorable outcome with a power of 90% and a 2-sided 5% alpha risk, assuming this rate would be 50% in the control arm.

has been corrected to:

The study was then powered to detect a decrease from 50% to 37% of patients with very unfavorable outcome with a power of 80% and a 2-sided 5% alpha risk, assuming this rate would be 50% in the control arm.

In the “statistical methods” section of the manuscript, the passage:

The 1-year survival rate without major deterioration in QoL (main endpoint, defined as reporting of an extreme problem” level in 1 of the 5 dimensions studied) will be compared between both arms using a piecewise exponential model considering any censorship and the repeated nature of observations, prohibiting the use of conventional methods of analysis of censored data

has been corrected to:

The 1-year survival rate without major deterioration in QoL (main endpoint, defined as reporting of death or a severe to extreme problem” level in 1 of the 5 dimensions studied) will be compared between both arms using a piecewise exponential model considering any censorship and the repeated nature of observations, prohibiting the use of conventional methods of analysis of censored data.

The correction will appear in the online version of the paper on the JMIR Publications website on April 14, 2023, together with the publication of this correction notice. Because this was made after submission to PubMed, PubMed Central, and other full-text repositories, the corrected article has also been resubmitted to those repositories.

