# Supplementary material for: Impact of a Postintensive Care Unit Multidisciplinary Follow-up on the Quality of Life (SUIVI-REA): Protocol for a Multicenter Randomized Controlled Trial
Source: JMIR Res Protoc. 2022 May 9;11(5):e30496. doi: 10.2196/30496 (PMC9127649; doi:10.2196/30496)
Supplement: Multimedia Appendix 1 [file resprot_v11i5e30496_app1.docx]

**Supplementary table:** Amendments to study protocol

| **Amendments** | **Date** | **Content** |
| --- | --- | --- |
| **N°1** | 14/09/2012 | - Change of the Project Manager - Centers removal (Clermont - Ferrand and Saint Louis) – and center addition (Dijon) - Inclusion of Guillain Barré patients at Raymond Poincaré Hospital - Other changes concerning the monitoring schedule, assessments, quality of life questionnaire |
| **N°2** | 03/06/2013 | - Modification of the inclusion and non-inclusion criteria: the patients with metastasized cancer with a life expectancy of several years and patients with mild depressive syndrome are eligible |
| **N°3** | 21/01/2014 | - Changes in inclusion criteria for improving enrollment – Extension of time for the first patient’s assessment up to seven days after their inclusion~~. – Addition of 2 centers (Poissy et Chartres)~~ - Modification of the selection criteria to improve inclusions - Modification of the study outline at the level of the 1st follow-up visit - Addition of 2 centers (Poissy and Chartres) |
| **N°4** | 19/03/2014 | - Addition of 3 new centers (Bichat, Lariboisière, Louis Mourier) |
| **N°5** | 04/07/2014 | - Study extension (inclusion period will be 3 years and the full study duration 4 years) |
| **N°6** | 09/01/2015 | - Inclusion of confused patients (subject to the agreement of the relative or trusted person) before they give their consent to proceed - Inclusion of patients hospitalized for voluntary drug intoxication - Change of the Project Manager - Addition of consent for relatives |
| **N°7** | 16/06/2015 | - Opening of two centers (HEGP, Saint Louis) and closing of one center (Poissy) |
| **N°8** | 19/01/2016 | - Modification of the inclusion criteria: inclusion of multiple trauma patients without severe head trauma - The Glasgow score should not be an exclusion criterion (initial Glasgow deletion <8 of the non-inclusion criterion No. 2) - Third-party consent, specific to patients with Guillain Barré (included in Raymond Poincaré Hospital) should be used for the inclusion of patients with a physical inability to write (but able to understand) regardless of the pathology - Rewording an inclusion criterion to make it clearer "patient who received continuous invasive and / or non-invasive mechanical ventilation for 2 hours per day, more than three days" |
| **N°9** | 23/01/2017 | - Reduction in the number of patients to be included (520 instead of 600)  - 12-month extension of the inclusion period |
| **N°10** | 13/12/2018 | - Possibility of consultation or examination at another site belonging to the same hospital group with the same medical team   - Investigator change in two centers - Change of promoter's representatives |
